# Label-free two-photon imaging of mitochondrial activity in murine macrophages stimulated with bacterial and viral ligands

**DOI:** 10.1038/s41598-021-93043-9

**Published:** 2021-07-07

**Authors:** Christian Harry Allen, Duale Ahmed, Olivia Raiche-Tanner, Vinita Chauhan, Leila Mostaço-Guidolin, Edana Cassol, Sangeeta Murugkar

**Affiliations:** 1 Department of Physics, Carleton University, 1125 Colonel By Drive, Ottawa, ON K1S 5B6 Canada; 2 Department of Health Sciences, Carleton University, 1125 Colonel By Drive, Ottawa, ON K1S 5B6 Canada; 3 Consumer and Clinical Radiation Protection Bureau, Healthy Environments and Consumer Safety Branch, Health Canada, Ottawa, K1A 0K9 Canada; 4 Department of Systems and Computer Engineering, Carleton University, 1125 Colonel By Drive, Ottawa, ON K1S 5B6 Canada; 5 Centre for Infection, Immunity and Inflammation, University of Ottawa, 451 Smyth Road, Ottawa, ON K1H 8M5 Canada

**Keywords:** Fluorescence imaging, Cellular imaging, Imaging the immune system

## Abstract

Mitochondria are the metabolic hub of the cell, playing a central role in regulating immune responses. Dysfunction of mitochondrial reprogramming can occur during bacterial and viral infections compromising hosts’ immune signaling. Comparative evaluation of these alterations in response to bacterial and viral ligands can provide insights into a cell’s ability to mount pathogen-specific responses. In this study, we used two-photon excitation fluorescence (TPEF) imaging to quantify reduced nicotinamide adenine dinucleotide phosphate (NAD(P)H) and flavin adenine dinucleotide (FAD) levels in the cell and to calculate the optical redox ratio (ORR), an indicator of mitochondrial dysfunction. Analyses were performed on RAW264.7 cells and murine bone marrow derived macrophages (BMM) stimulated with bacterial (LPS) and viral (Poly(I:C)) ligands. Responses were cell type dependent, with primary cells having significantly higher levels of FAD and higher oxygen consumption rates suggesting BMM may be more dependent on mitochondrial metabolism. Our findings also suggest that FAD-TPEF intensity may be a better predictor of mitochondrial activity and localization since it demonstrates unique mitochondrial clustering patterns in LPS vs. Poly(I:C) stimulated macrophages. Collectively, we demonstrate that TPEF imaging is a powerful label-free approach for quantifying changes in mitochondrial function and organization in macrophages following bacterial and viral stimuli.

## Introduction

Macrophages serve as important sentinels against infection, recognizing viruses and bacteria, initiating inflammatory responses, and recruiting other immune cells to the site of infection. After a microorganism has been cleared, they also remove apoptotic-derived debris and promote tissue repair and remodeling to re-establish homeostasis^1–4^. Over the last decade, several studies have shown that cellular metabolism plays a central role in regulating macrophage function^5–7^. The mitochondria, often called the “powerhouses of the cell”, regulate several immune processes^8^. In response to bacterial ligands, such as lipopolysaccharide (LPS), mitochondrial function is reprogrammed (i.e., decreased oxidative phosphorylation [OXPHOS]) to support reactive oxygen species (ROS) production, which is required to support robust inflammatory responses^9–12^. Alternatively, in response to viruses or synthetic viral ligands such as Polyinosinic:polycytidylic acid (Poly(I:C)), sustained OXPHOS activity and increased ROS production are required to mount functional antiviral immune responses^13–15^. Collectively, these studies suggest that mitochondria might play a central role in modulating pathogen-specific responses. Additional research is required to fully understand these processes.

To date, many studies evaluating the effects of mitochondria on macrophage function have been performed using in vitro model systems. These include primary murine bone marrow-derived macrophages (BMM) and human monocyte-derived macrophages (MDM) or cell lines such as murine RAW 264.7 and human THP-1 cell lines^9,16–18^. BMM and MDM are the models of choice as they are generally considered more representative of in vivo responses. However, their responses are heterogenous, the number of available cells can be limiting, and they can be difficult to genetically modify. Cell lines, on the other hand, lack genetic variation, proliferate at high levels and are easy to culture and genetically modify^19–21^. In order to use cell lines as representative models, we must validate that they undergo the same features of metabolic reprogramming.

A variety of methods are currently used to investigate mitochondrial bioenergetics in immune cells, including assessing oxygen consumption rate (OCR), mitochondrial membrane potential and mitochondrial NAD(P)H levels^13,22–24^. While informative, these methods evaluate collective responses in the total cell population or require significant manipulation of the cells to perform single cell analyses (e.g., detachment of cells for flow cytometry), which can affect cellular metabolism^25,26^. Single cell imaging-based approaches have the advantage of capturing the dynamics and heterogeneity of metabolic activity with minimal manipulation. Two-photon excited fluorescence (TPEF) imaging is a nonlinear optical imaging technique that has high (~ 0.5 µm) spatial resolution with inherent 3D sectioning capability and can be performed in a label-free manner, without the addition of external image contrast agents^27^. TPEF imaging of endogenous fluorophores, such as the reduced form of nicotinamide adenine dinucleotide and its phosphorylated counterpart (NAD(P)H) as well as flavin adenine dinucleotide (FAD) offers a non-invasive and label-free approach for quantitative metabolic imaging of cells and tissue^28–30^. The optical redox ratio (ORR) defined as the ratio of the TPEF intensity of FAD and the sum of the TPEF intensity of FAD and NAD(P)H^28,29,31^, or alternately defined as the ratio of the TPEF intensities of FAD and NAD(P)H^30^, has been used to evaluate cellular redox status. Changes in the ORR and the NAD(P)H fluorescence lifetime also correspond to changes in the rate of glucose catabolism compared to the rate of oxidative phosphorylation^28,32,33^. Recent studies have also used NAD(P)H-TPEF images to evaluate structural changes in the mitochondrial organization in terms of the dynamic fusion or fission^34–36^. These metrics, implemented independently or in combination, have been demonstrated to be powerful tools for quantifying the mitochondrial activity in cells and tissue for example, in cancer cells^32,37^ and their response to treatment^33,38^, and in tissue engineering and stem cell differentiation^39,40^.

In this work, we demonstrate a novel application of TPEF imaging in evaluating single cell mitochondrial reprogramming in macrophages stimulated with bacterial (LPS) and viral (Poly(I:C)) ligands. We also evaluate how these responses differ between murine BMM and immortalized RAW264.7 cells. Changes in response were quantified using the ORR as well as, the FAD-TPEF and NAD(P)H-TPEF intensities between treatments for each cell type and between RAW264.7 and BMM cells for each treatment group. Additionally, we evaluated alterations in mitochondrial organization using a Fourier transform-based approach applied to the NAD(P)H-TPEF images. NADH makes up the majority of the NADH/NADPH pool, and thus NAD(P)H fluorescence signal, within macrophages^23^. For the first time, we demonstrate the application of a metric based on the distances between FAD-rich structures in the FAD-TPEF images, as a measure of the amount of mitochondrial clustering in the macrophages following stimulation with bacterial and viral ligands. In addition, we show that the distance of the FAD-rich structures relative to the centre (nucleus) of the cell, as well as the spatial correlation between the FAD-TPEF and NAD(P)H-TPEF intensities in each cell, can provide valuable new insights into the potential mechanisms by which mitochondria contribute to pathogen-specific responses.

## Methods

### Culturing of primary mouse macrophages and mouse macrophage cell lines

RAW264.7 cells (Sigma) were initially thawed and cultured in DMEM supplemented with 20% (v/v) of fetal bovine serum (FBS) and 1% (v/v) penicillin/streptomycin (Life Technologies). After the first three passages, cells were maintained in complete media with 10% FBS. All animal procedures were approved by the Carleton University Animal Care Committee and were conducted in accordance with the guidelines provided by the Canadian Council for Animal Care. The study was carried out in compliance with the ARRIVE guidelines. Bone marrow cells, isolated from the tibias and femurs of 6–13-week old C57BL/6 mice, were differentiated in DMEM media with 10% (v/v) fetal bovine serum (FBS), 1% (v/v) penicillin/streptomycin (Life Technologies), and 15% L929 fibroblast cell-conditioned medium on a 100 mm Petri dish as previously described^41^. After 10 days of differentiation, the resulting primary mouse BMM were detached, counted and plated according to experimental design.

### Assessing the mitochondrial energetic profiles of RAW 264.7 cells and primary BMM via extracellular flux analysis

RAW264.7 cells and primary mouse BMM were seeded onto Seahorse XFp cell culture miniplates at 50,000 cells/well (Seahorse Bioscience) and allowed to rest in a CO_2_ incubator for 3 h and overnight respectively prior to experimentation. Cells were then stimulated with 100 ng/mL LPS or 10 ng/mL Poly(I:C) for 18 h before evaluating mitochondrial function using the Seahorse XFp Cell Mito Stress Test Kit (Agilent). OCR measurements were recorded after consecutive injections of oligomycin (OM), carbonyl cyanide-p-trifluoromethoxyphenylhydrazone (FCCP), and mixture of rotenone and antimycin A (ROT/AA). Changes in OCR were used to measure the spare respiratory capacity percentage (SRC) and ATP production of activated cells.

### TPEF image acquisition

TPEF imaging was performed on a multiphoton microscopy platform utilizing a femtosecond laser (Insight DS+, Spectra Physics) as the light source. The tunable (680–1300 nm) output with 120 fs pulses was coupled to an open frame, custom-built laser-scanning microscope^42^. A 60X water immersion microscope objective (NA = 1.1, Olympus) was used to focus the excitation light onto the sample and to collect the back-scattered (epi−) TPEF signal in non-descanned mode using a multialkali photomultiplier tube operating at room temperature (H9305-03, Hamamatsu). The laser was tuned to 720 nm or 880 nm for excitation of the NAD(P)H or FAD fluorescence respectively. Each acquisition took approximately 68 s and the time between acquiring at 720 nm and 880 nm was typically less than 60 s. This varied depending on how quickly the 880 nm beam could be attenuated to the correct power using a 1% power tap. The TPEF signal was detected using a 447/60 nm bandpass filter for NAD(P)H or a 535/70 nm bandpass filter for FAD. TPEF images from rhodamine B diluted in methanol (5.8 × 10^–4^ g/mL) were recorded during each data set using the 535/70 nm BP filter, one at the 720 nm excitation and the other at the 880 nm excitation. Rhodamine TPEF images were used for normalization of the NAD(P)H and FAD images^30^. ScanImage (Version 5.6, Vidrio Technologies)^43^ was used for control of the laser scanning and image acquisition.

RAW264.7 cells and primary mouse BMM were plated onto 35 mm glass bottom petri dishes (MatTek Life Sciences), having a 10 mm microwell and a No. 1.5 glass bottom coverslip at a density of 250,000 cells per dish and allowed to rest overnight. Both cell types were either left unstimulated or treated with either 100 ng/mL LPS or 10 ng/mL Poly(I:C) for 18 h prior to imaging. Images (90 RAW264.7, 76 BMM) were acquired over four data sets with each data set including TPEF from 1 dish of control cells, 1 dish of Poly(I:C)-treated cells, 1 dish of LPS-treated cells and 1 Rhodamine dish for normalizing cell images. About 6–7 regions of interest (ROIs) containing approximately 3–7 cells each, were imaged per dish. For the RAW264.7 cells, the number of cells imaged was 152 for the control, 129 for the LPS, and 132 for the Poly(I:C) treatment. For the BMM, a total of 127 cells were imaged for the control, 96 cells for the LPS, and 134 cells for the Poly(I:C) treatment. The laser power at the sample was 10 mW and 20 mW for the 720 nm and 880 nm excitation, respectively. This choice of laser power along with averaging over 20 frames provided the optimum signal to noise ratio in the images without causing any photodamage to the cells. Images were acquired over a ~ 75 µm × 75 µm field of view using an 8.6 µs pixel dwell time at 512 × 512 resolution and 16-bit depth.

### Image analysis

#### Optical redox ratio (ORR)

Coordinates of single cells in TPEF images, were manually selected using ImageJ^44^. TPEF cell images were then preprocessed (see supplementary materials) using three main steps: segmentation using Otsu thresholding^45^, background correction, and rhodamine normalization. The ORR was calculated for each individual cell, using

1 $$\begin{array}{c}ORR=\frac{FAD}{FAD+NAD(P)H}\end{array}$$

where FAD and NAD(P)H are the corresponding TPEF signal intensities for a given pixel respectively. This definition of ORR was used since it preserves the normal distribution in the data and provides values bound between 0 and 1^29^. This generates an “ORR Image”, from which the mean ORR of that cell is found from the mean pixel intensity of all pixels within the cell.

The mean intensity over cell area for FAD-TPEF and NAD(P)H-TPEF images was calculated and compared between treatments for each cell type. These calculations were repeated for each treatment to make comparisons between RAW264.7 and BMM cells, in order to further explore potential differences in metabolic activity across cell types. Intensity values are image values (16-bit range) normalized to 0–1 range. Mean intensities greater than 1.5 interquartile ranges above the upper quartile or below the lower quartile were considered outliers and removed from the analysis, which removed 1.7%, 4.5%, and 1.2% of the FAD, NADH, and ORR values respectively from the RAW264.7 set, and 7.8%, 2%, and 6.2% from the BMM set.

#### Spatial correlation between NAD(P)H and FAD TPEF signals for individual cells

The Pearson correlation coefficient, given by

2 $$\begin{array}{c}{\rho }_{x,y}=\frac{\frac{1}{n}{\sum }_{i}^{n}\left[\left({x}_{i}-{\mu }_{x}\right)\left({y}_{i}-{\mu }_{y}\right)\right]}{{\sigma }_{x}{\sigma }_{y}}\end{array}$$

where ${\mu }_{x}$ and ${\mu }_{y}$ are the mean values of the two sets being compared, and ${\sigma }_{x}$ and ${\sigma }_{y}$ are their standard deviations, was applied to each cell using the corresponding intensity values from the FAD-TPEF and NAD(P)H-TPEF images. Only pixels containing NAD(P)H or FAD signals were considered. For each cell, we obtained a correlation score which was then assessed for each treatment. Gaussian blurred images were used for this measurement to reduce the effect of noise, and only pixels containing NAD(P)H or FAD signal were considered. Further evidence supporting our interpretation of the spatial correlation results was found using Manders’ coefficients^46^, which are the colocalized fraction of a given signal with respect to another signal from a two channel (two fluorophore) fluorescent image. For example, the Manders’ coefficient for NAD(P)H for a given cell would be the fraction of the entire NAD(P)H-TPEF intensity that is colocalized with FAD in that cell:

3 $$\begin{array}{c}{M}_{NAD\left(P\right)H}=\frac{\sum {I}_{NAD\left(P\right)H,col}}{\sum {I}_{NAD\left(P\right)H}}\end{array}$$

where the numerator is the sum of NAD(P)H-TPEF pixel intensities for pixels that also have FAD-TPEF signal, and the denominator is the sum of all NAD(P)H-TPEF pixels intensities, for that cell.

#### Mitochondrial organization

##### Power spectral density analysis of NAD(P)H-TPEF images

Fourier analysis of NAD(P)H-TPEF images is a well-established approach for evaluating mitochondrial organization^34,35,39,47^. In this approach, the squared amplitude of the 2D Fourier transform of the NAD(P)H-TPEF image is computed pixel-wise. This is radially averaged to obtain the power spectral density (PSD) of an image as a function of the radial spatial frequency $k$. PSD is thus a measure of the prevalence of spatial features of different length scales in the image. The PSD is fit with an inverse power law proportional to ${k}^{-\beta }.$ For biological cells, the exponent $\beta$ has values between 0 and 4 with higher values of $\beta$ corresponding to a more fragmented mitochondrial network consisting of clusters of high intensity. In this work, NAD(P)H-TPEF images were pre-processed (see Supplementary Material) to remove low-intensity regions of the image attributed to the background and the weakly-fluorescent nuclei, as well as to remove artifacts due to the effect of cell and nuclei borders^35^. The PSD was fit with a power law over the spatial frequency range of 0.1–0.5 µm^−1^ corresponding to spatial features that are between 2 and 10 µm in size, associated with the approximate range of lengths of mitochondria in murine macrophages^48^. The absolute value of the slope of the linear fit of the PSD plot provided the $\beta$ value for mitochondrial clustering.

#### Object identification and centre of mass (CM) identification for FAD-TPEF images

Following background correction, the number of objects (or, in this study, FAD-rich structures) was defined by setting a threshold on the image. All pixels for which the intensity is above the established background level (I > 0) were considered as object’s pixels whilst other pixels are considered as background^49^. A map with all pixels associated to objects is created and the number of objects as well as the area is obtained from such a map as shown in Figure S1 in Supplementary Materials. Based on the pixel coordinates of each identified object, the geometrical centre for each object is identified based on such coordinates. The analysis was performed in steps and visually inspected after each step. First, we identified the CM of each cell. Next, we identified the CM of each FAD-rich structure. Finally, by overlaying the CM maps to the optical images, it was possible to identify the structures part of each individual cell (CM and their respective FAD-rich structures).

#### Statistical analysis

A D’Agostino-Pearson and Shapiro–Wilk normality test was used to assess the data, specifically mean ORR, FAD-TPEF, NAD(P)H-TPEF, *β*, and FAD-TPEF structural distance distributions. While some single distributions (e.g., Mean ORR Control set for RAW264.7 cells) were found to be normal, many were not, especially distributions associated with LPS treated cells. In particular, the distance distributions of FAD-rich structures to cell centre of mass and between FAD-rich structures for RAW264.7 cells and primary BMM were found to follow a lognormal distribution. The normality test results can be found in Tables S1 and S2 in the Supplementary Materials. Since normality could not be assumed with this data, the nonparametric two-tailed Mann–Whitney U test was used to assess significance of differences between distribution medians. This was performed using GraphPad Prism version 6.0. Data are shown as mean ± SEM; p < 0.05 was considered significant, and all two-tailed Mann–Whitney U tests had greater than 0.90 power.

## Results

### Primary macrophages have higher levels of respiration and mitochondrial ATP production

**Figure 1:**
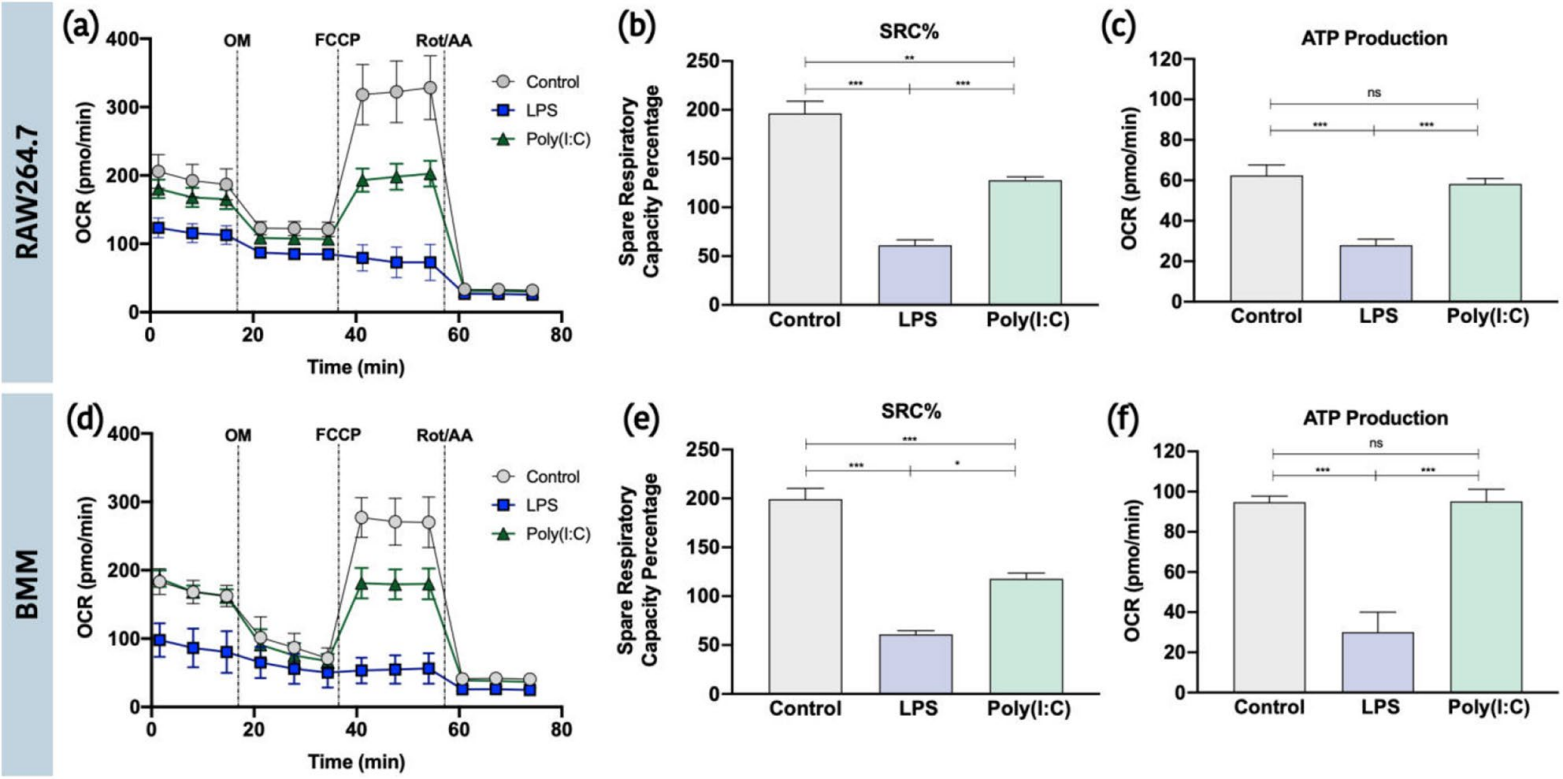
Primary macrophages are more dependent on mitochondrial-derived ATP than macrophages originating from cell lines. RAW264.7 cells (**a**–**c**) and primary mouse BMM (**d**–**f**) were seeded onto Seahorse XFp miniplates and were treated with 100 ng/mL LPS or 10 ng/mL Poly(I:C) for 18 h. Mitochondrial function was measured following sequential injections of Oligomycin (OM), Carbonyl cyanide-p-trifluoromethoxyphenylhydrazone (FCCP), and rotenone plus antimycin A (Rot/AA), resulting in the calculation of the spare respiratory capacity percentage (SRC%) and ATP production. Data represents mean ± SEM of three individual mice (BMM) or experiments (RAW) (*p < 0.05, **p < 0.01, and ***p < 0.001).

To set a baseline of mitochondrial activity, we first evaluated changes in OXPHOS activity in RAW264.7 cells and BMM stimulated with LPS and Poly(I:C) using the Seahorse XFp extracellular efflux analyzer. Changes in OCR were assessed following consecutive doses of OM, FCCP and ROT/AA and were used to calculate differences in spare respiratory capacity percentage (SRC%) and mitochondrial ATP production (Fig. 1). Consistent with previous studies^6,13,22,50^, LPS stimulation of both RAW264.7 and BMM cells is associated with a severe loss in basal respiration (BMM: ↓50%; RAW264.7: ↓40%), ATP production (BMM: ↓68%; RAW264.7: ↓55%), and SRC (BMM: ↓69%; RAW264.7: ↓69%) compared to untreated cells. Alternatively, Poly(I:C) stimulation did not alter basal respiration and ATP production but was associated with a significant loss in SRC (BMM: ↓41%; RAW264.7: ↓35%). Of note, respiration in untreated and Poly(I:C) stimulated BMM resulted in significantly more mitochondrial-derived ATP compared to RAW264.7 cells (p < 0.001). These results suggest that RAW264.7 cells, which are highly proliferative, may be less dependent on OXPHOS for energy production^16^ and that cell lines vs. primary cells may differ in their prioritization of mitochondrial function***.***

### Optical redox ratio (ORR) is sensitive to changes in oxygen consumption only in LPS-treated RAW264.7 cells

**Figure 2:**
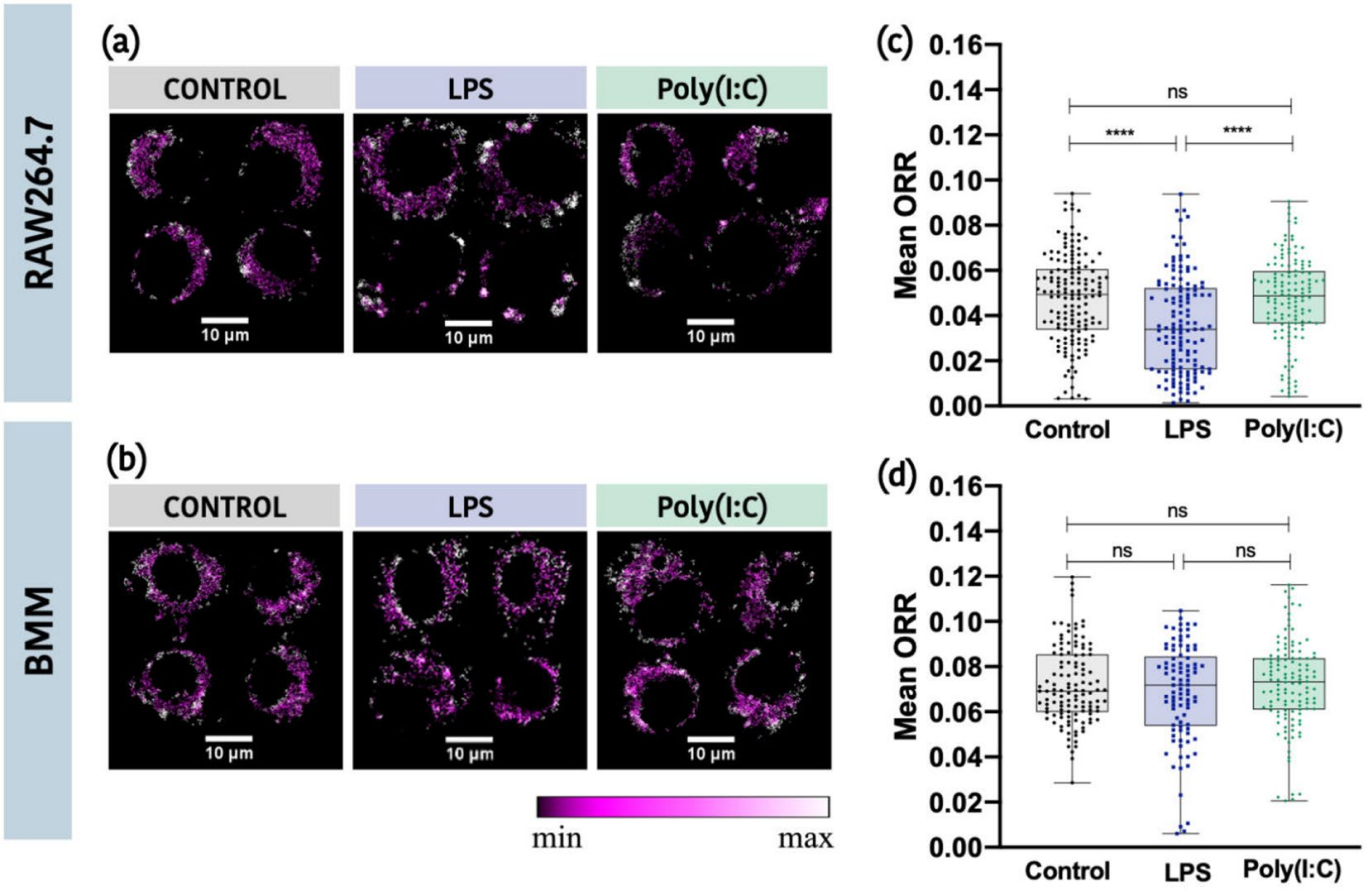
Mean ORR is significantly lower in LPS treated cells than that observed in control and Poly(I:C) treated cells for RAW264.7 cells but not BMM. Representative ORR images of cells from different regions of interest for control, LPS-treated and Poly(I:C)-treated for RAW264.7 cells (**a**) and BMM cells (**b**). Mean ORR values for control, LPS, and Poly(I:C) treated cells for RAW264.7 cells (**c**) and BMM cells (**d**) (*p < 0.05, **p < 0.01, ***p < 0.001, ****p < 0.0001).

Next, we evaluated changes in mitochondrial metabolism using FAD-TPEF and NAD(P)H-TPEF mean intensities and calculated the ORR. We found that the mean ORR in LPS treated RAW264.7 cells was significantly lower than that observed in control and Poly(I:C) treated cells (Fig. 2a,c). However, these differences were not observed in BMM cells (Fig. 2b,d). These differences may be due, in part, to the heterogenous nature of single cell responses in BMM compared to RAW264.7 cells. This heterogeneity may limit the use of the ORR in BMM.

We then examined differences in FAD-TPEF and NAD(P)H-TPEF mean intensities in RAW264.7 cells and BMM (Figure S2 in Supplementary Materials). Consistent with the Seahorse analysis, LPS stimulation of both RAW264.7 cells and BMM was associated with a decreased accumulation of FAD signal, likely reflecting a decrease in OXPHOS activity. We also found that Poly(I:C) stimulation of BMM but not RAW264.7 cells was associated with an increased accumulation of NAD(P)H. While it is unclear what is driving this increase, it highlights the differential modulation of cellular metabolism in primary cells vs. cell lines.

**Figure 3:**
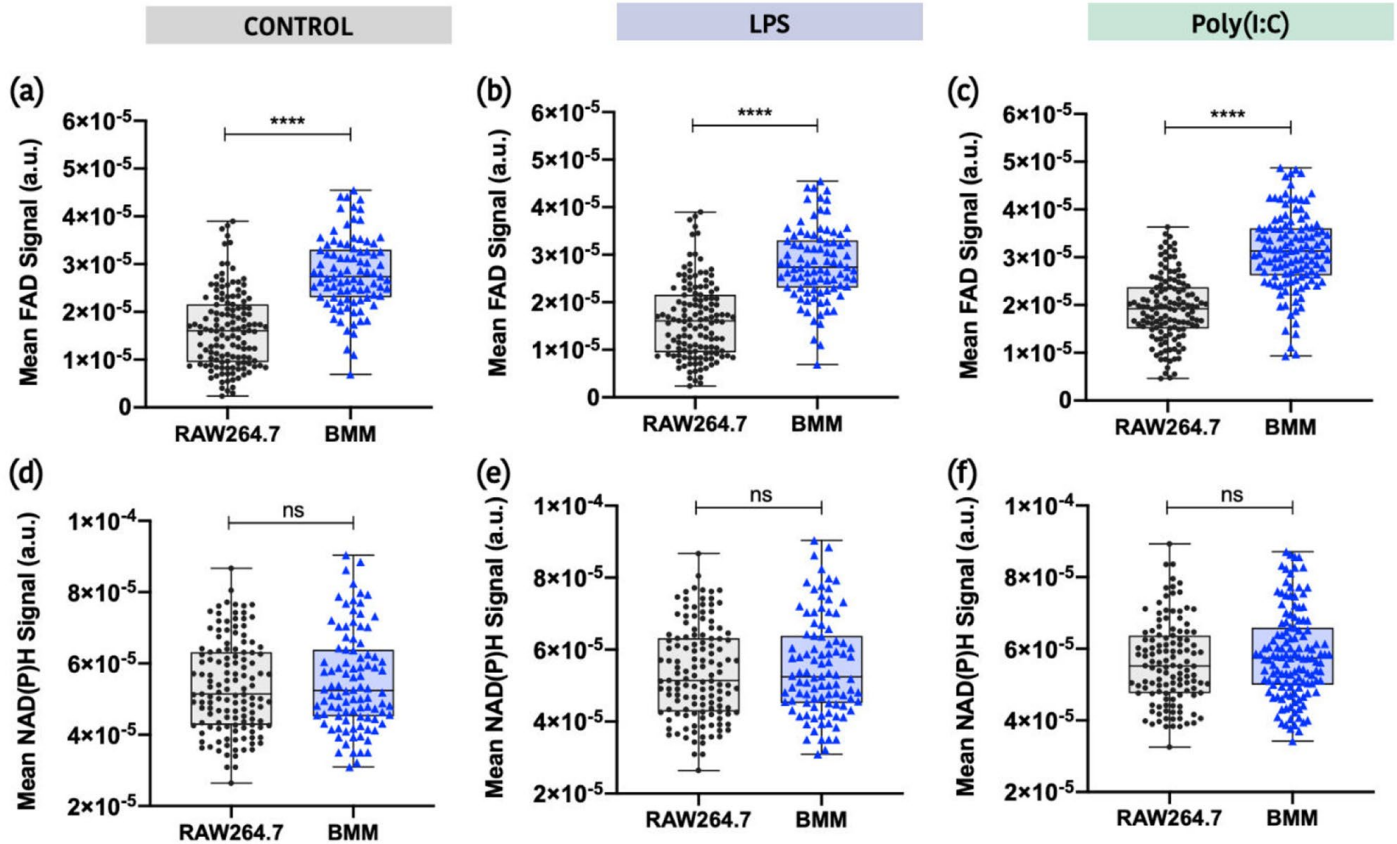
Comparison of FAD-TPEF intensities and NAD(P)H-TPEF intensities between cell types for each treatment group. (**a**–**c**) Mean FAD-TPEF intensities evaluated for RAW264.7 cells and BMM for each treatment group (Control, LPS, and Poly(I:C)). (**d**–**f**) Mean NAD(P)H-TPEF intensities evaluated for RAW264.7 cells and BMM for each treatment group (*p ≤ 0.05, **p ≤ 0.01, ***p ≤ 0.001, ****p ≤ 0.0001).

To further explore potential differences in metabolic activity across cell types, we compared FAD-TPEF intensities and NAD(P)H-TPEF intensities in RAW264.7 and BMM cells (Fig. 3). While both cell types had similar mean NAD(P)H levels, BMM had a significantly higher mean FAD levels across all treatment types. Consistent with the OCR data, these results suggest an increased requirement for mitochondrial metabolism in BMM.

### Spatial correlation between NAD(P)H and FAD TPEF signals is significantly different between cell treatments

**Figure 4:**
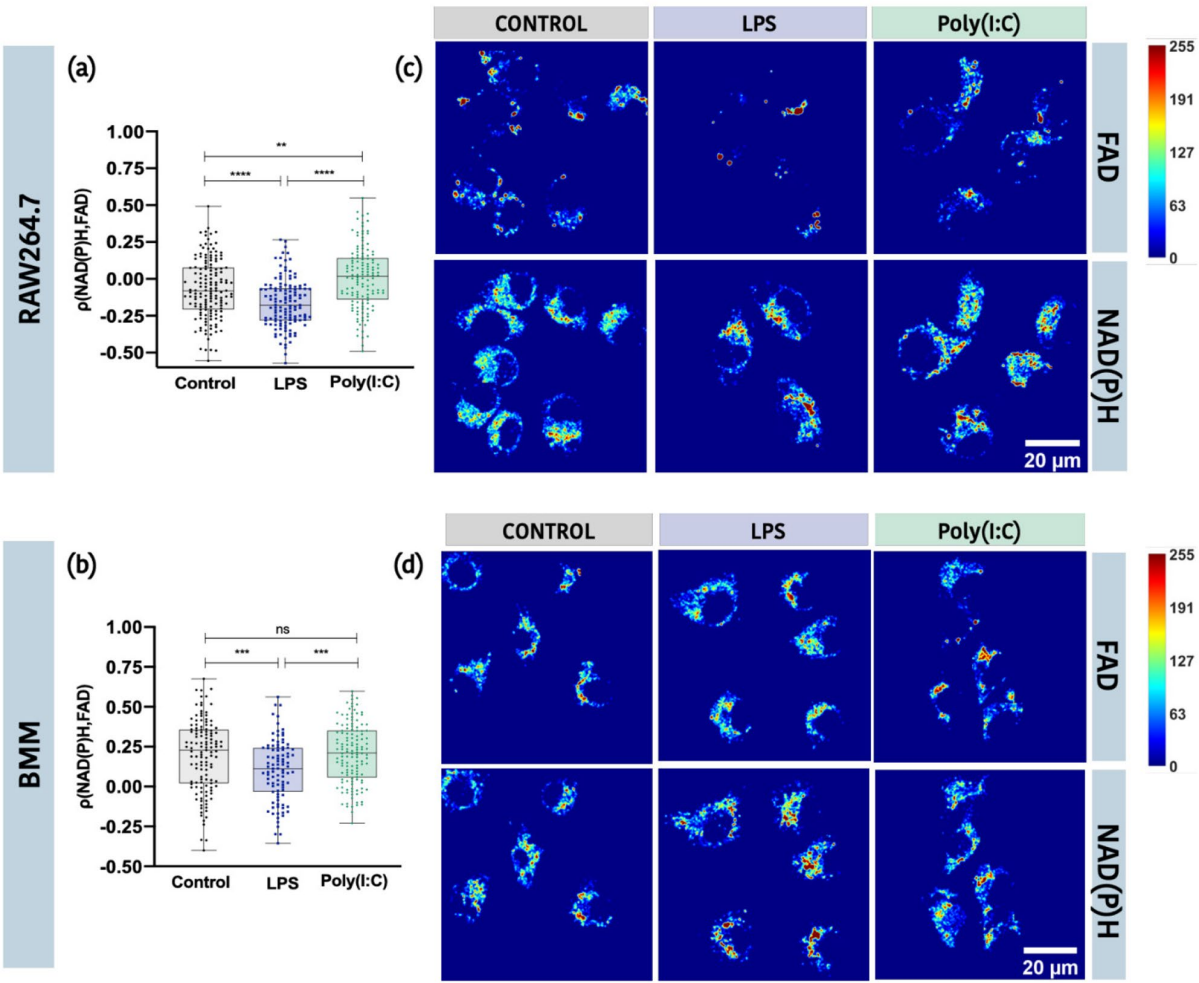
Analysis of the spatial correlation between the FAD-TPEF and NAD(P)H-TPEF intensities in each cell. Correlation between FAD-TPEF and NAD(P)H-TPEF intensities for Control, LPS, and Poly(I:C) treated cells for (**a**) RAW264.7 cells and (**b**) BMM cells (*p ≤ 0.05, **p ≤ 0.01, ***p ≤ 0.001, ****p ≤ 0.0001). Representative images of RAW264.7 cells (Top panel (**c**)) and primary BMM (Bottom panel (**d**)) shown in a thermal colour palette. In each panel, the top row is FAD channel and bottom row is NAD(P)H channel with Control, LPS and Poly(I:C) treated cells from left to right.

Next, we evaluated if there was an association between the spatial localization of FAD-TPEF and NAD(P)H-TPEF signals using Pearson’s correlation analysis (Fig. 4). Overall, we found that the association between FAD-TPEF and NAD(P)H-TPEF signal was highly heterogeneous across individual cells and that these associations changed following LPS and Poly(I:C) stimulation (Supplemental Table S3). Approximately 36% of unstimulated RAW264.7 cells showed some positive correlation between the two signals. However, the mean correlation coefficient was close to zero (− 0.07 ± 0.20) suggesting most cells had no association between FAD and NAD(P)H levels. In contrast, 78% of unstimulated BMM had some level of correlation between FAD-TPEF and NAD(P)H-TPEF signals. The mean correlation coefficient was only weakly positive (0.194 ± 0.22) suggesting a high level of heterogeneity in individual cell responses. Interestingly, compared to the unstimulated cells, LPS-stimulation was associated with an overall increase in the number of cells with negative correlations between FAD-TPEF and NAD(P)H-TPEF, with a more pronounced increase of ~ 20% in RAW264.7 cells compared to ~ 6% in BMM cells. Further analysis of the Manders’ coefficients showed this negative correlation was associated with limited colocalization and not an anti-correlation of NAD(P)H-TPEF and FAD-TPEF signals (Supplemental Figure S3) in LPS-stimulated RAW264.7 cells as compared to unstimulated and Poly(I:C)-stimulated RAW264.7 cells. While we cannot exclude the possibility that this correlation maybe driven by altered levels of NAD(P)H and FAD in the mitochondria, we instead believe this correlation likely reflects the increased glycolytic activity seen in LPS-stimulated cells and the accumulation of NAD(P)H from glycolysis in the cytosol^51^. On the other hand, Poly(I:C)-stimulated RAW264.7 cells had the largest increase of ~ 19% in the number of cells with positive correlations between FAD and NAD(P)H signal (Supplemental Table S3). This is consistent with the results of increased colocalization of FAD-TPEF and NAD(P)H-TPEF intensities in Poly(I:C)-stimulated RAW264.7 cells as compared to unstimulated RAW264.7 cells (Supplemental Figure S3) obtained from the Manders’ coefficients, suggesting a reprioritization/increased dependency on mitochondrial activity in these cells. This increase was not observed in BMM, which likely reflects the more significant dependence of BMM on mitochondrial activity in both Poly(I:C)-stimulated and unstimulated cells.

### FAD-TPEF intensity localization is a potential marker for assessing differences in mitochondrial organization

**Figure 5:**
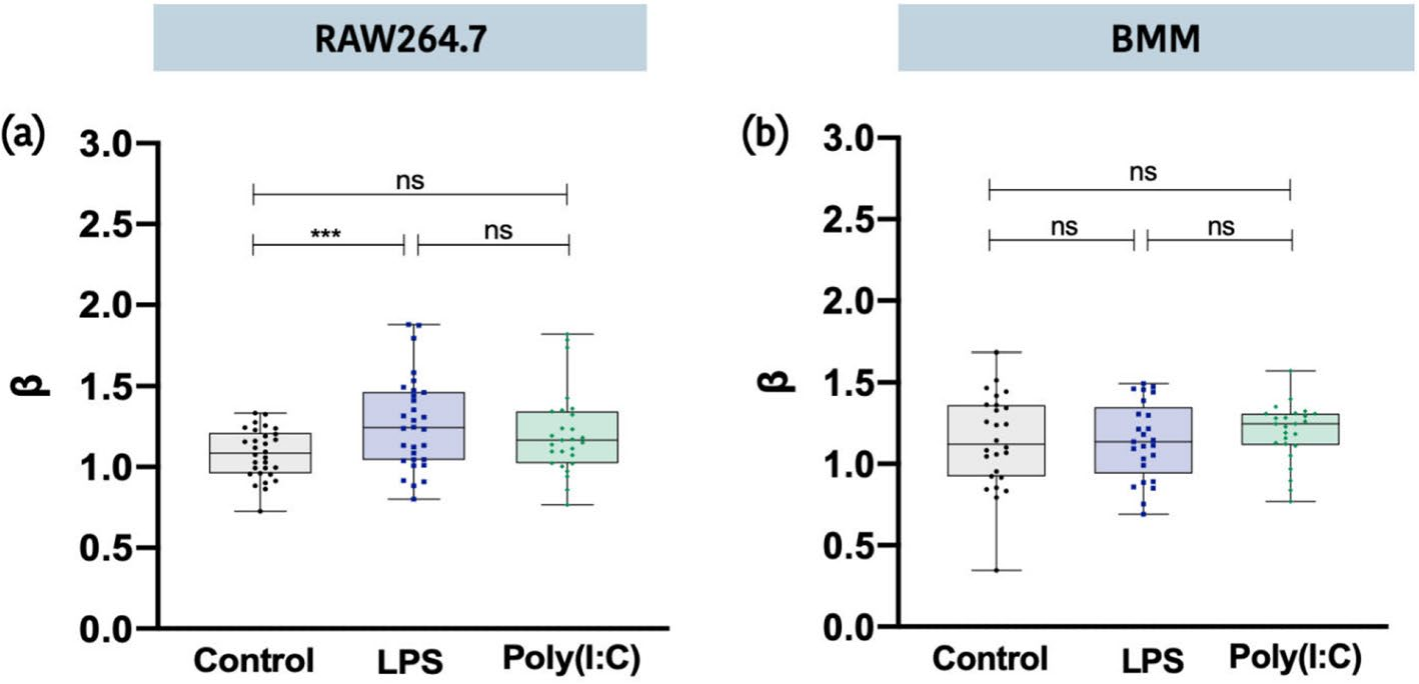
PSD analysis of NAD(P)H-TPEF images to evaluate mitochondrial organization. β values determined from the slope of the power law fit of the radially averaged PSD as a function of spatial frequency of NAD(P)H-TPEF full field images for (**a**) RAW264.7 cells and (**b**) BMM cells (*p ≤ 0.05, **p ≤ 0.01, ***p ≤ 0.001, ****p ≤ 0.0001).

Finally, we used the NAD(P)H-TPEF images to evaluate changes in mitochondrial organization in RAW264.7 cells and BMM stimulated with LPS and Poly(I:C). As previously described, NAD(P)H-TPEF images were used to calculate a mitochondrial clustering value *β*. This value has been used to evaluate mitochondrial clustering patterns across a range of cell types where increased *β* values have been linked to more fragmented mitochondrial networks^28,34,39^ and higher levels of glycolysis^39^. Interestingly, we found that LPS stimulation in RAW264.7 cells but not BMM was associated with an increase in the *β* value (Fig. 5). While both cell types increase glycolytic activity in responses to LPS^13,16,52,53^, it is unclear why we did not detect any changes in the β value in primary BMM. It may be related to the overall metabolic activity of each cell type. Unlike RAW264.7 cells, which are transformed proliferating cells, BMM are terminally differentiated primary cells with lower biosynthetic requirements.

**Figure 6:**
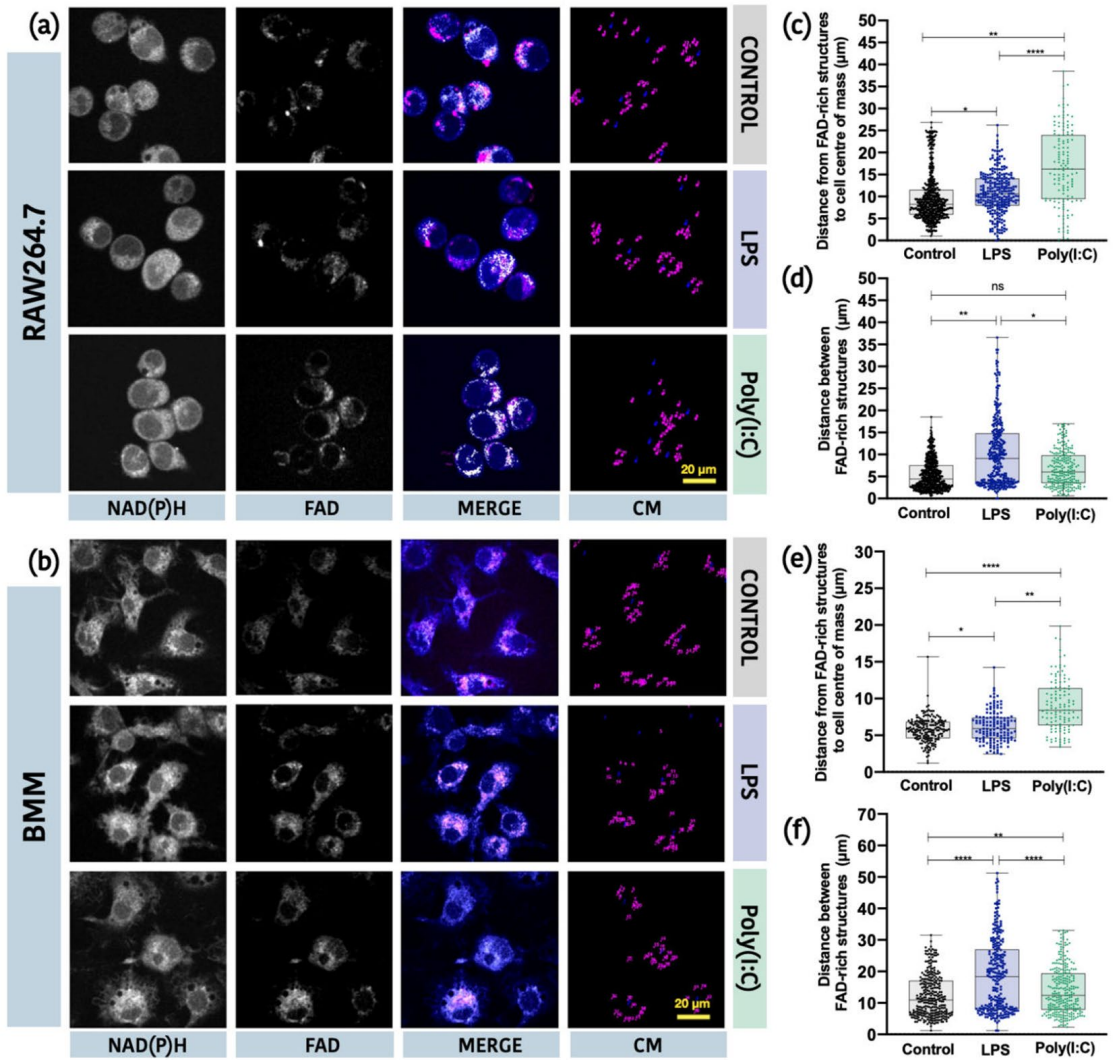
FAD-TPEF intensity localization is a potential marker for mitochondrial organization. Representative images of (**a**) RAW264.7 cells and (**b**) primary BMM, showing from left to right, NAD(P)H-TPEF intensity, FAD-TPEF intensity, Merged TPEF intensity from NAD(P)H and FAD channels, and the centre of mass (CM) distribution of FAD-rich structures. Distance of FAD-rich structures to cell centre of mass for RAW264.7 cells (**c**) and primary BMM (**e**). Distance between FAD-rich structures for RAW264.7 cells (**d**) and primary BMM (**f**) (*p < 0.05, **p < 0.01, ***p < 0.001, ****p < 0.0001, ns: non-significant).

Given the limited utility of the *β* value in our primary cell model, we determined if FAD-TPEF images could be used to assess alterations in mitochondrial organization. To do this, we evaluated changes in the distance of FAD-rich structures from the centre of the cell (e.g., perinuclear localization) as well as the distance between FAD-rich structures in the cytosol (e.g., distribution throughout the cell). Upon spatial identification of FAD-TPEF signal, we found that FAD-rich structures in control and LPS-stimulated RAW264.7 cells and primary BMM were more closely associated with the centre of the cell (nuclei) (Fig. 6). However, the distance between FAD-rich structures was significantly larger suggesting more fragmentation/compartmentalization of the individual structures. In contrast, FAD-rich structures were located farther from the nucleus following Poly(I:C) treatment of RAW264.7 cells and primary BMM, and the distance between these structures was less diffuse as compared to the FAD-rich structures in control and LPS-stimulated RAW264.7 cells and primary BMM. Descriptive statistics for the distance of FAD-rich structures from cell CM and from each other can be found in Tables S4 and S5 of the Supplementary Material, respectively. Collectively, these results suggest that LPS stimulation is associated with mitochondrial network fragmentation and perinuclear accumulation. Alternatively, Poly(I:C) stimulation is associated with cytosolic accumulation of mitochondria in fused networks that support sustained OXPHOS activity.

## Discussion

Increasing evidence suggests that mitochondria play a central role in regulating pathogen-specific immune responses in macrophages but the mechanisms underlying these processes remain incompletely understood. To better characterize these processes, there is a need for (1) cell models that are as representative of responses in vivo and (2) methodologies that can evaluate mitochondrial distribution and function in live cells with single cell resolution. Here, we used TPEF imaging to evaluate changes in mitochondrial function in live macrophage cell lines and primary cells. A summary of our results based on the analysis of multiple parameters is presented in Table S6 in Supplementary Materials. We found that mitochondrial reprogramming is highly heterogeneous across individual cells and that changes observed in macrophage-like cells lines are not necessarily representative of those observed in primary cells. We found that FAD-TPEF intensity localization is a potential marker to map changes in mitochondrial function and organization in murine macrophages.

TPEF imaging has been used in various systems to evaluate changes in mitochondrial function in health and disease but its use in immune cells has been somewhat limited^48,54^. In cancer cells, ORR is a metric of cellular redox status and has been shown to correlate with flux analyzer results^28,55^. Outcomes of this study showed that changes in ORR (Fig. 2) in RAW264.7 cells but not BMM were consistent with changes in oxygen consumption rates following LPS stimulation (Fig. 1). Despite a near complete loss of OXPHOS activity in BMM stimulated with LPS, this was not reflected by the ORR. We speculate that these differences may be related to heterogeneity of metabolic reprogramming across cell types. RAW264.7 are immortalized cell lines that grow fast and have a doubling time of approximately 11 h in standard culture conditions^56^. These cells are highly glycolytic and dependent almost exclusively on glycolysis for energy production and to mount inflammatory responses^16^. On the other hand, BMM are terminally differentiated cells with different metabolic demands^13^. While the overall trends in FAD intensity were similar in RAW264.7 and BMM following stimulation (Figure S2), we found BMM have significantly higher levels of FAD compared to RAW264.7 cells (Fig. 3) and are more dependent on OXPHOS activity for ATP production (Fig. 1). This may be attributed to differences in mitochondrial abundance and dynamics in these cells and suggests primary cells may be more dependent on mitochondria and less dependent on glycolysis to become functionally activated. Interestingly, Pan et al. identified distinct proteomic features between primary hepatocytes and the hepatocyte cell line Hepa1-6, which included a loss in mitochondrial abundance and reduced levels of proteins related to oxidative phosphorylation and fatty acid metabolism in the cell line^20^. Conversely, proteins related to glycolysis were up-regulated in Hepa1-6 cells highlighting the functional adaptation cell lines may undergo to survive under in vitro cell culture conditions.

NAD(P)H-TPEF has been a useful tool in evaluating mitochondrial organization^28,29^. In these studies, Fourier-based analysis of the NAD(P)H-TPEF intensity images are used to calculate *β* values. Elevated *β* values have been linked to more fragmented mitochondrial networks^28,39,34^ and increased glycolysis^39^. Here, we found that *β* values increased in LPS stimulated RAW264.7 cells but not BMM (Fig. 5). Again, we speculate that these differences may be associated with the increased glycolytic activity of this cell line. Consistent with this hypothesis, we found that LPS-stimulation of RAW264.7 cells was associated with an increase in the number of cells with negative correlations between the spatial location of FAD-TPEF and NAD(P)H-TPEF intensities (Table S3 and Fig. 4), suggesting poor colocalization of these signals (Figure S3), likely the result of more NAD(P)H accumulating in the cytosol. This agrees with earlier work^54^ which identified a significant decrease in the NAD(P)H bound fraction in murine macrophages as determined from NAD(P)H fluorescence lifetime imaging (FLIM) measurements that were analyzed using the phasor approach^28,29,57^. A similar reduction in the NAD(P)H-bound fraction has been reported in hypoxic HFK cells^28^. Based on these findings we believe that analyzing the Pearson correlation coefficient of the spatial location of the FAD-TPEF and NAD(P)H-TPEF intensities inside the same cell could be used as another metric to retrieve the relative information of the free to bound fraction of NAD(P)H. Future work will investigate the use of FLIM analysis of the NAD(P)H-TPEF and FAD-TPEF decay profiles in RAW264.7 cells and BMM to compare with these results.

Instead of NAD(P)H-TPEF intensities, we found that FAD-TPEF signal was a better predictor of changes in mitochondrial activity in macrophage like cell lines and primary cells. FAD is used by various energy pathways such as the tricarboxylic acid (TCA) cycle, OXPHOS, fatty acid oxidation (FAO), and branched chain amino acid catabolism and is primarily found in the mitochondria. Specifically, we found LPS stimulation was associated with increased mitochondrial network fragmentation as measured by distance between FAD-rich structures (Fig. 6). Pouli et al. have reported that mitochondrial clustering typically represents fragmented mitochondrial organizations that are required to optimize energy production within the cell^36^. We also found these FAD-rich structures in LPS stimulated cells were closely associated with the nucleus (Fig. 6). Further, Al-Mehdi et al. have reported that perinuclear clustering of mitochondria results in ROS accumulation in the nucleus, which can alter transcriptional complex assembly and gene transcription^58^. Based on these studies, we believe that mitochondria may adopt this fragmented perinuclear clustering in LPS stimulated cells to support increased ROS production and rapidly transmit these bioactive molecules into the nucleus to promote inflammatory signaling. In contrast to LPS, Poly(I:C) stimulation was associated with accumulation of highly organized FAD-rich structures within the cytosol. We hypothesize that these cytosolic networks are required to sustain OXPHOS activity and mitochondrial ATP production, which are required to mount functional antiviral immune responses^13,15^. Thus, the distance of the FAD-rich structures from the cell centre presents another useful metric associated with mitochondrial translocation occurring in relation to the immune signaling function of the mitochondria^58^.

In conclusion, the work presented here provides evidence that TPEF imaging is a robust label-free method to examine cellular mitochondrial activity in immune cells such as macrophages. For the first time, TPEF imaging allowed for single cell-level assessment of mitochondrial activity showing heterogeneous responses following stimulation with viral and bacterial insults, which may have direct implications for the functional role of mitochondrial reprogramming in driving these pathogen specific responses. Our study demonstrated the application of novel metrics based on the FAD-TPEF intensity localization to quantify the changes in cellular metabolism. This work also suggests that when studying mitochondrial function and reprogramming, it is important to carefully evaluate the model system being used for the study. Additionally, other methods for assessing particle colocalization^59,60^ can be explored aiming to obtain a broader collection of metrics and move towards defining nonparametric methods for quantitative interpretation.

## Supplementary Information

Supplementary Information.
