# Supplementary material for: Label-free two-photon imaging of mitochondrial activity in murine macrophages stimulated with bacterial and viral ligands
Source: Sci Rep. 2021 Jul 7;11:14081. doi: 10.1038/s41598-021-93043-9 (PMC8263786; doi:10.1038/s41598-021-93043-9)
Supplement: Supplementary file 1 — Supplementary Information. [file 41598_2021_93043_MOESM1_ESM.docx]

**Label-free two-photon imaging of mitochondrial activity in murine macrophages stimulated with bacterial and viral ligands**

**Supplementary Information**

Christian Harry Allen^1^, Duale Ahmed^2^, Olivia Raiche-Tanner^1^, Vinita Chauhan^3^, Leila Mostaço-Guidolin^4^, Edana Cassol^2,5^ and Sangeeta Murugkar^1^

^1^Department of Physics, Carleton University, 1125 Colonel By Drive, Ottawa, ON, Canada K1S 5B6

^2^Department of Health Sciences, Carleton University, 1125 Colonel By Drive, Ottawa, ON, Canada K1S 5B6

^3^Consumer and Clinical Radiation Protection Bureau, Healthy Environments and Consumer Safety Branch, Health Canada, Ottawa, Canada K1A 0K9

^4^Department of Systems and Computer Engineering, Carleton University, 1125 Colonel By Drive, Ottawa, ON, Canada K1S 5B6

^5^Centre for Infection, Immunity and Inflammation, University of Ottawa, 451 Smyth Road, Ottawa, ON, Canada, K1H 8M5

Correspondence and requests for materials should be addressed to:

E.C. ([edana.cassol@carleton.ca](mailto:edana.cassol@carleton.ca)) or S.M. (smurugkar@physics.carleton.ca)

***Image Pre-processing***

TPEF cell images were preprocessed using MATLAB (MathWorks) in three main steps: segmentation, background correction, and rhodamine normalization. Cells were segmented from background in the images by creating a binary mask using Otsu thresholding^[[1]](#endnote-2)^ on the sum of the Gaussian blurred NAD(P)H-TPEF and FAD-TPEF images. This mask was used to define the background region and find its mean, which was subtracted from the whole image. Any negative pixels or pixels not belonging to a cell region were then set to zero. Rhodamine TPEF images were flattened using a 5^th^ order polynomial surface fit, and averaged. This average and surface fit was used to normalize the NAD(P)H-TPEF and FAD-TPEF cell images acquired for the corresponding excitation wavelength, for each treatment type, correcting for changes in intensity between sample sets and correcting for the intensity gradient of the FOV.

Final processing was performed cell by cell. Considering no NAD(P)H-TPEF and FAD-TPEF signals are supposed to be detected within the nucleus region, two iterations of Otsu thresholding were performed in order to suppress any remaining background autofluorescence originated from sources other than NAD(P)H and FAD molecules, and then segment each cell body based only on NAD(P)H-TPEF and FAD-TPEF signals.

***Object identification and centre of mass identification for FAD TPEF images***

The number of objects (or, in this study, FAD-rich structures) was defined by setting a threshold on the image. All pixels for which the intensity is above the established background level (I>0) were considered as object’s pixels whilst other pixels are considered as background^[[2]](#endnote-3)^. A map with all pixels associated to objects is created and the number of objects as well as the area is obtained from such a map. This can be seen in Figure S1 below. Based on the pixel coordinates of each identified object, the geometrical centre for each object is identified based on such coordinates.


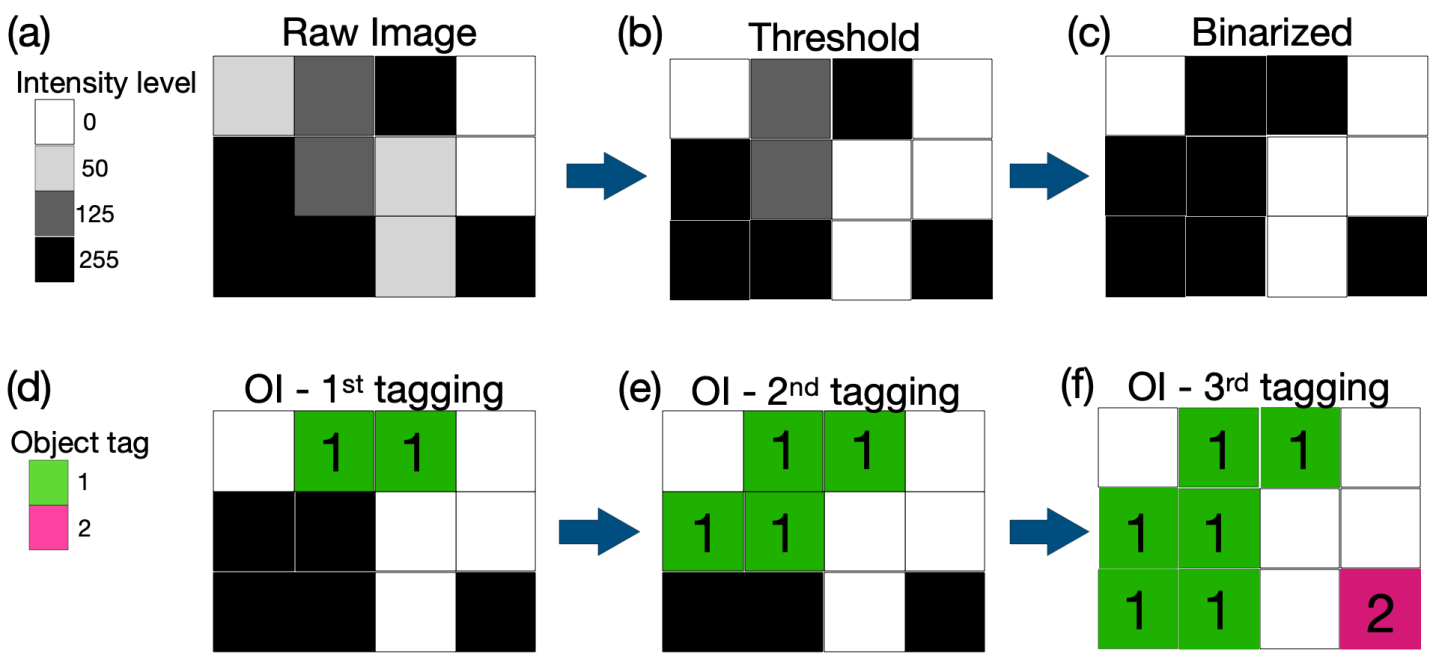


Figure S1: Summary of the process of object identification: (a) original image; (b) threshold definition; (c) binarized image; (d) a tag is given to the first object’s pixel found, tagging of the first line; (e) tagging of the second line; (f) tagging of the third line. Upon completion of screening all image lines, the algorithm accounts for the number of tags used. OI: object identification.

***Mean intensities for FAD-TPEF and NAD(P)H-TPEF cell images***

The distributions of FAD-TPEF and NAD(P)H-TPEF intensities are given in Figure S2 for both RAW 264.7 cells and BMM.


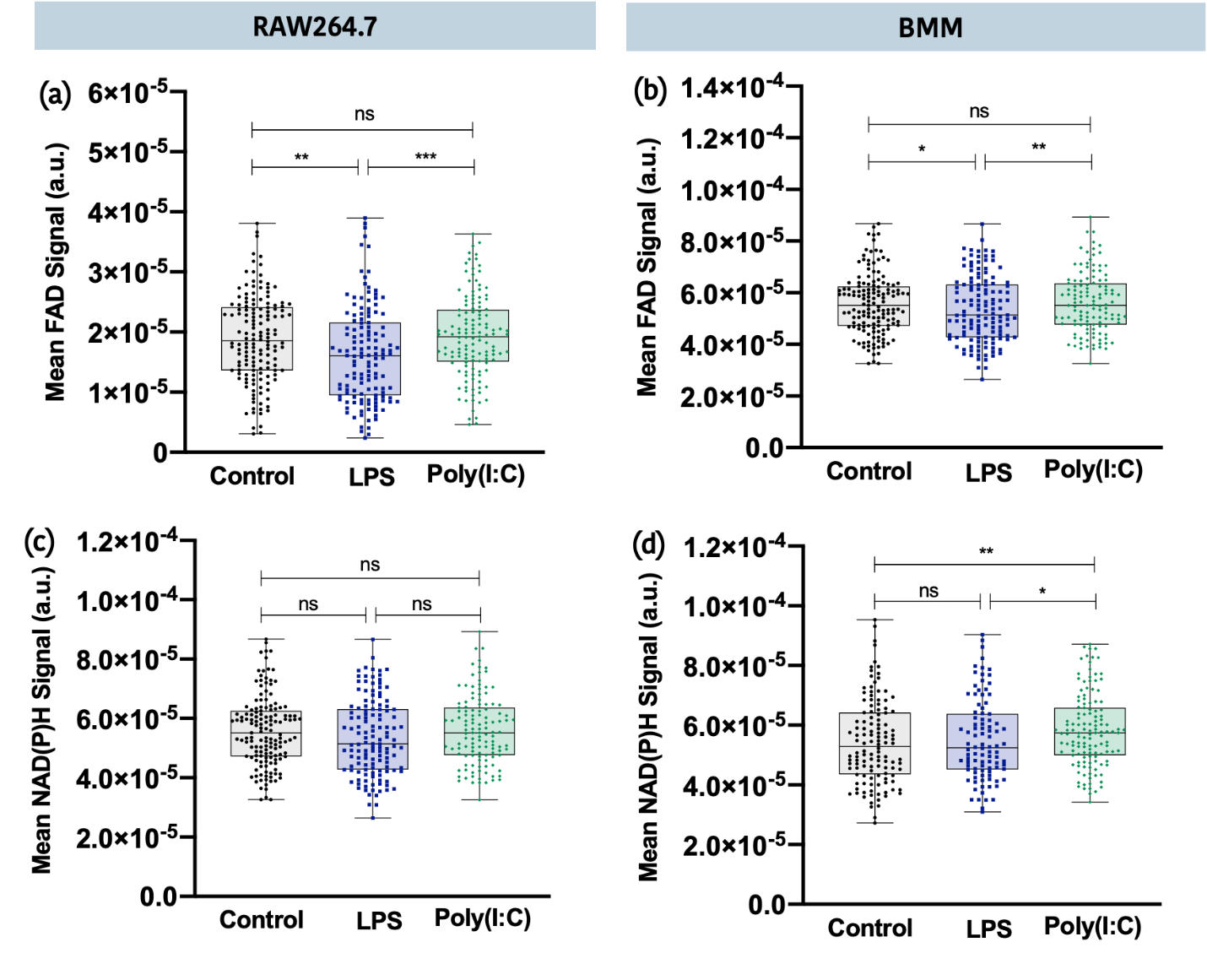


Figure S2: Differences in FAD-TPEF and NAD(P)H-TPEF mean intensities in RAW264.7 cells and BMM. Mean FAD-TPEF intensities for control, LPS, and Poly(I:C) treated cells for (a) RAW264.7 cells and (b) BMM cells and mean NAD(P)H-TPEF intensities for (c) RAW264.7 and (b) BMM cells. (* p ≤ 0.05, ** p ≤ 0.01, *** p ≤ 0.001, **** p ≤ 0.0001)

***Normality test results for all data sets***

Table S1 – Normality test p values associated with mean FAD-TPEF and NAD(P)H-TPEF intensity distributions, mean ORR value distributions shown in Figure 2, and Beta values associated with Figure 5.

|  | | | **Control** | **LPS** | **Poly(I:C)** |
| --- | --- | --- | --- | --- | --- |
|  |  |  |  |  |  |
| RAW264.7 | Mean ORR | D'Agostino & Pearson test | 0.6278 | 0.0231 | 0.3850 |
|  |  | Passed normality test (alpha=0.05)? | Yes | No | Yes |
|  |  | Shapiro-Wilk test | 0.4431 | 0.0006 | 0.0662 |
|  |  | Passed normality test (alpha=0.05)? | Yes | No | Yes |
|  | Mean FAD | D'Agostino & Pearson test | 0.6917 | 0.0171 | 0.5466 |
|  |  | Passed normality test (alpha=0.05)? | Yes | No | Yes |
|  |  | Shapiro-Wilk test | 0.5759 | 0.0010 | 0.3968 |
|  |  | Passed normality test (alpha=0.05)? | Yes | No | Yes |
|  | Mean NADH | D'Agostino & Pearson test | 0.1656 | 0.0104 | 0.1264 |
|  |  | Passed normality test (alpha=0.05)? | Yes | No | Yes |
|  |  | Shapiro-Wilk test | 0.0372 | 0.0227 | 0.0455 |
|  |  | Passed normality test (alpha=0.05)? | No | No | No |
|  | Beta | D'Agostino & Pearson test | 0.6919 | 0.4220 | 0.0506 |
|  |  | Passed normality test (alpha=0.05)? | Yes | Yes | Yes |
|  |  | Shapiro-Wilk test | 0.6890 | 0.2612 | 0.0294 |
|  |  | Passed normality test (alpha=0.05)? | Yes | Yes | No |
| BMM | Mean ORR | D'Agostino & Pearson test | 0.1139 | 0.0051 | 0.1508 |
|  |  | Passed normality test (alpha=0.05)? | Yes | No | Yes |
|  |  | Shapiro-Wilk test | 0.0365 | 0.0010 | 0.0523 |
|  |  | Passed normality test (alpha=0.05)? | No | No | Yes |
|  | Mean FAD | D'Agostino & Pearson test | 0.1656 | 0.0104 | 0.1264 |
|  |  | Passed normality test (alpha=0.05)? | Yes | No | Yes |
|  |  | Shapiro-Wilk test | 0.0372 | 0.0227 | 0.0455 |
|  |  | Passed normality test (alpha=0.05)? | No | No | No |
|  | Mean NADH | D'Agostino & Pearson test | 0.0398 | 0.0564 | 0.0797 |
|  |  | Passed normality test (alpha=0.05)? | No | Yes | Yes |
|  |  | Shapiro-Wilk test | 0.0106 | 0.0059 | 0.0090 |
|  |  | Passed normality test (alpha=0.05)? | No | No | No |
|  | Beta | D'Agostino & Pearson test | 0.2591 | 0.4452 | 0.2227 |
|  |  | Passed normality test (alpha=0.05)? | Yes | Yes | Yes |
|  |  | Shapiro-Wilk test | 0.5258 | 0.3356 | 0.1359 |
|  |  | Passed normality test (alpha=0.05)? | Yes | Yes | Yes |

Table S2 – Normality test p values associated with FAD structural distance data presented in Figure 6.

|  | | | **Control** | **LPS** | **Poly(I:C)** |
| --- | --- | --- | --- | --- | --- |
| RAW264.7 | Distance from FAD-rich structures to cell centre of mass | D'Agostino & Pearson test | <0.0001 | 0.0426 | 0.0240 |
|  |  | Passed normality test (alpha=0.05)? | No | No | No |
|  |  | Shapiro-Wilk test | <0.0001 | 0.0175 | 0.1131 |
|  |  | Passed normality test (alpha=0.05)? | No | No | Yes |
|  | Distance between FAD-rich structures | D'Agostino & Pearson test | <0.0001 | <0.0001 | 0.0002 |
|  |  | Passed normality test (alpha=0.05)? | No | No | No |
|  |  | Shapiro-Wilk test | <0.0001 | <0.0001 | <0.0001 |
|  |  | Passed normality test (alpha=0.05)? | No | No | No |
| BMM | Distance from FAD-rich structures to cell centre of mass | D'Agostino & Pearson test | <0.0001 | 0.0026 | 0.0192 |
|  |  | Passed normality test (alpha=0.05)? | No | No | No |
|  |  | Shapiro-Wilk test | <0.0001 | 0.0028 | 0.0027 |
|  |  | Passed normality test (alpha=0.05)? | No | No | No |
|  | Distance between FAD-rich structures | D'Agostino & Pearson test | <0.0001 | <0.0001 | <0.0001 |
|  |  | Passed normality test (alpha=0.05)? | No | No | No |
|  |  | Shapiro-Wilk test | <0.0001 | <0.0001 | <0.0001 |
|  |  | Passed normality test (alpha=0.05)? | No | No | No |

***Summary of spatial correlation results between FAD-TPEF and NAD(P)H-TPEF images***

The correlation distributions plotted in Figure 4 of the paper are summarized in Table S3 below, in which mean values, standard deviations, min and max values, and the positive fraction (in percent) of the correlation between FAD-TPEF and NAD(P)H-TPEF cell images.

Table S3: Mean correlation, standard deviation, and min and max correlation values for RAW264.7 cells and primary BMM cells, stimulated with LPS and Poly(I:C).

|  | **Correlation** | **Control** | **LPS** | **Poly(I:C)** |
| --- | --- | --- | --- | --- |
| **RAW** | Mean | -0.066 | -0.165 | 0.007 |
|  | Std Dev | 0.200 | 0.164 | 0.210 |
|  | Min | -0.555 | -0.571 | -0.490 |
|  | Max | 0.493 | 0.266 | 0.548 |
|  | % Positive^a^ | 35.5 | 16.3 | 53.8 |
| **BMM** | Mean | 0.194 | 0.101 | 0.206 |
|  | Std Dev | 0.223 | 0.193 | 0.187 |
|  | Min | -0.401 | -0.356 | -0.231 |
|  | Max | 0.676 | 0.562 | 0.598 |
|  | % Positive^a^ | 78.0 | 71.9 | 82.1 |

^a^ % Positive denotes the percentage of cells with positive correlation between the NAD(P)H-TPEF and FAD-TPEF signals

***Analysis of colocalization of FAD-TPEF and NAD(P)H-TPEF intensities using Manders’ coefficients***

In order to better determine whether poor colocalization was responsible for the more negative correlation of FAD-TPEF and NAD(P)H-TPEF intensities for LPS treated cells, we measured the Manders’ coefficients^[[3]](#endnote-4)^, which are the fraction of a given fluorescence intensity that is colocalized with another fluorophore, meaning the fraction of intensity that comes from pixels that show intensity from both fluorophores. The results for FAD-TPEF and NAD(P)H-TPEF, for both RAW264.7 and BMM sets, are given in Figure S3 below.


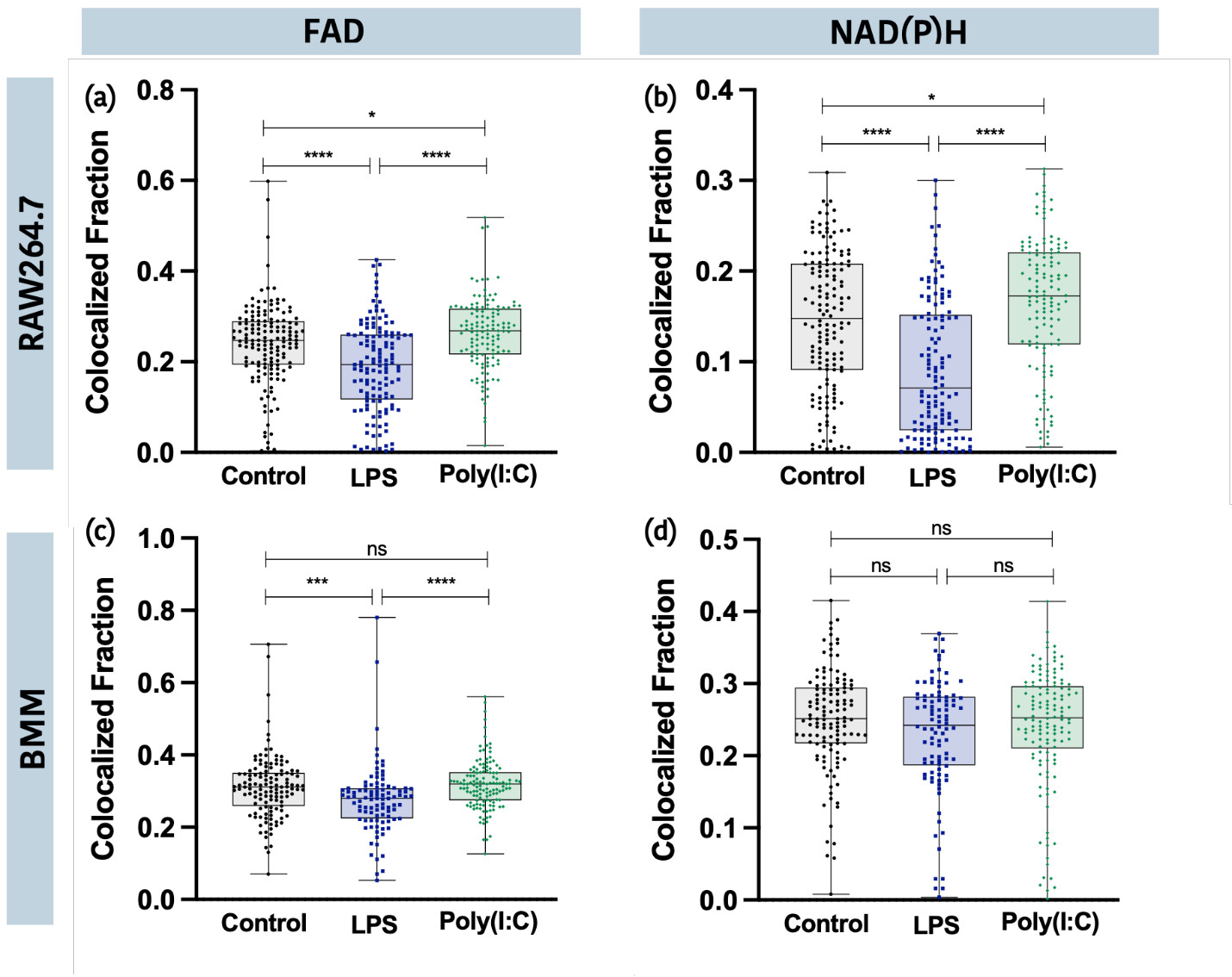


Figure S3: The Manders’ coefficients are the fraction of colocalized signal for two overlapping fluorescent signals, in this case FAD and NAD(P)H for RAW264.7 cells (a-b) and BMM (c-d).

***Summary statistics of FAD structural distances***

Table S4 – Descriptive statistics corresponding to distances between cells CM to FAD-rich structures

|  |  | **Control** | **LPS** | **Poly(I:C)** |
| --- | --- | --- | --- | --- |
| **RAW264.7** | Number of values | 365 | 264 | 133 |
|  | Minimum | 1.0 | 0.25 | 0.13 |
|  | 25% percentile | 5.9 | 8.0 | 9.4 |
|  | Median | 8.2 | 11 | 16 |
|  | 75% percentile | 12 | 14 | 24 |
|  | Maximum | 27 | 26 | 38 |
| **BMM** | Number of values | 190 | 139 | 119 |
|  | Minimum | 1.2 | 2.5 | 3.4 |
|  | 25% percentile | 4.6 | 4.6 | 6.4 |
|  | Median | 5.8 | 5.9 | 8.4 |
|  | 75% percentile | 6.8 | 7.4 | 11 |
|  | Maximum | 16 | 14 | 20 |

Table S5 – Descriptive statistics associated to distances between cells FAD-rich structures

to FAD-rich structures

|  |  | **Control** | **LPS** | **Poly(I:C)** |
| --- | --- | --- | --- | --- |
| **RAW264.7** | Number of values | 580 | 308 | 228 |
|  | Minimum | 0.5 | 0.7 | 0.59 |
|  | 25% percentile | 2.5 | 3.7 | 3.5 |
|  | Median | 4.4 | 9.1 | 6.0 |
|  | 75% percentile | 7.5 | 15 | 9.8 |
|  | Maximum | 19 | 37 | 17 |
| **BMM** | Number of values | 303 | 267 | 279 |
|  | Minimum | 1.2 | 1.2 | 2.3 |
|  | 25% percentile | 6.6 | 8.1 | 7.8 |
|  | Median | 11 | 18 | 12 |
|  | 75% percentile | 17 | 27 | 19 |
|  | Maximum | 32 | 51 | 33 |

***Power spectral density analysis of NAD(P)H-TPEF images***

The power spectral density (PSD) of an image is the absolute value squared (pixel by pixel) of the 2D fast Fourier transformed (FFT) image. The radially averaged PSD of NAD(P)H-TPEF cell images has been shown^[[4]](#endnote-5),^^[[5]](#endnote-6),^^[[6]](#endnote-7),^^[[7]](#endnote-8)^ to follow a decaying power law given by

$Ak^{-\beta},$ (1)

where $k$ is the radial spatial frequency. Thus, by taking the log of the radially averaged PSD vs the log of k, the slope of the linear fit will provide the exponent $-\beta$. The absolute value of $\beta$ has been used in past studies^4-7^ as a measure of mitochondrial organization, with significant differences in average $\beta$ between different cell types (e.g. cancerous vs healthy^4,6^) or treatments (e.g. change after irradiation^7^). This method involves a pre-processing step referred to as “digital object cloning” or “clone stamping”^5^, that is performed on the background-corrected images. In this step, regions of an image with no signal are filled in with signal containing parts. This is done by randomly shifting the image and regions with signal to any dark pixels, in order to avoid spatial frequency effects of cell and nuclear boundaries on the PSD. In our work, the digital object cloning followed with finding $\beta$ from PSD was performed on full, multi-cell NAD(P)H images of the RAW264.7 and BMM cells after applying two rounds of Otsu thresholding to remove unwanted autofluorescence attributed to the background and the weakly-fluorescent nuclei. The digital object cloning was performed ten times per frame and the mean absolute value of each FFT image was used to generate the PSD to find the $\beta$ values used in Fig. 6.

The power-law exponent β is a fractal metric indicating whether a pattern exhibits fractional Gaussian noise (0 < β <2), fractional Brownian noise (2 < β < 4), or non-fractal white Gaussian noise for β = 0^5^. For bound NAD(P)H, β can be thought of as corresponding to the correlation in mitochondrial structure within a cell, and in the fractional Brownian regime, a higher β corresponds to increased fragmentation of the mitochondrial network i.e. increased clustering^5^.

The following two figures, Figure S4 and S5, show some sample intermediate images and a plot of the β fit (log-log) in the process of extracting β values from full region NAD(P)H-TPEF images.


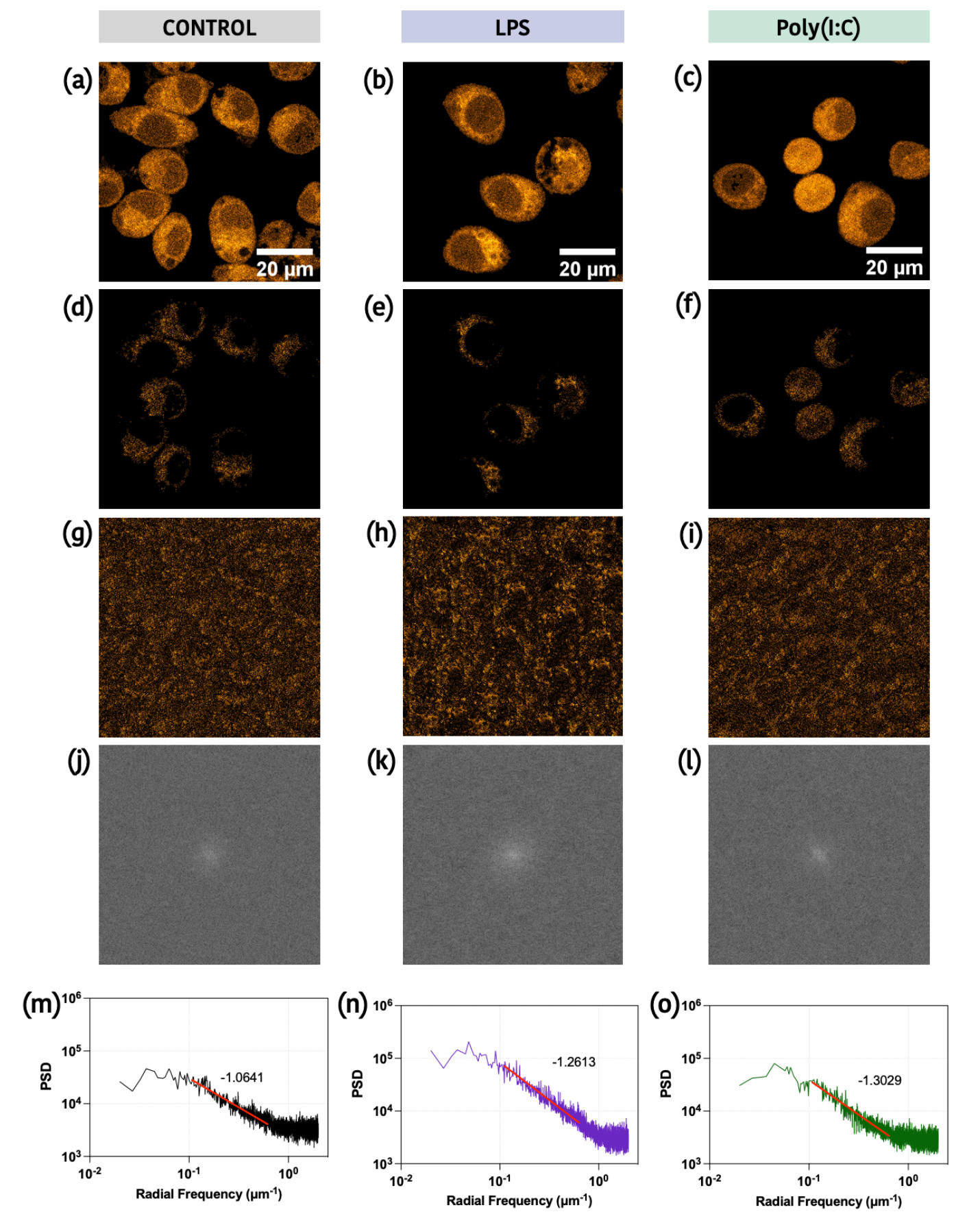


Figure S4: PSD analysis for RAW264.7 cells. Representative NAD(P)H-TPEF images (a-c) after thresholding is applied (d-f), after clone-stamping (g-i), and corresponding 2D Fourier transformed images (j-l). PSD spectra as a function spatial frequencies (m-o) for Control (a,d,g,j,m), LPS treated (b,e,h,k,n) and Poly(I:C) treated (c,f,i,l,o) cells.


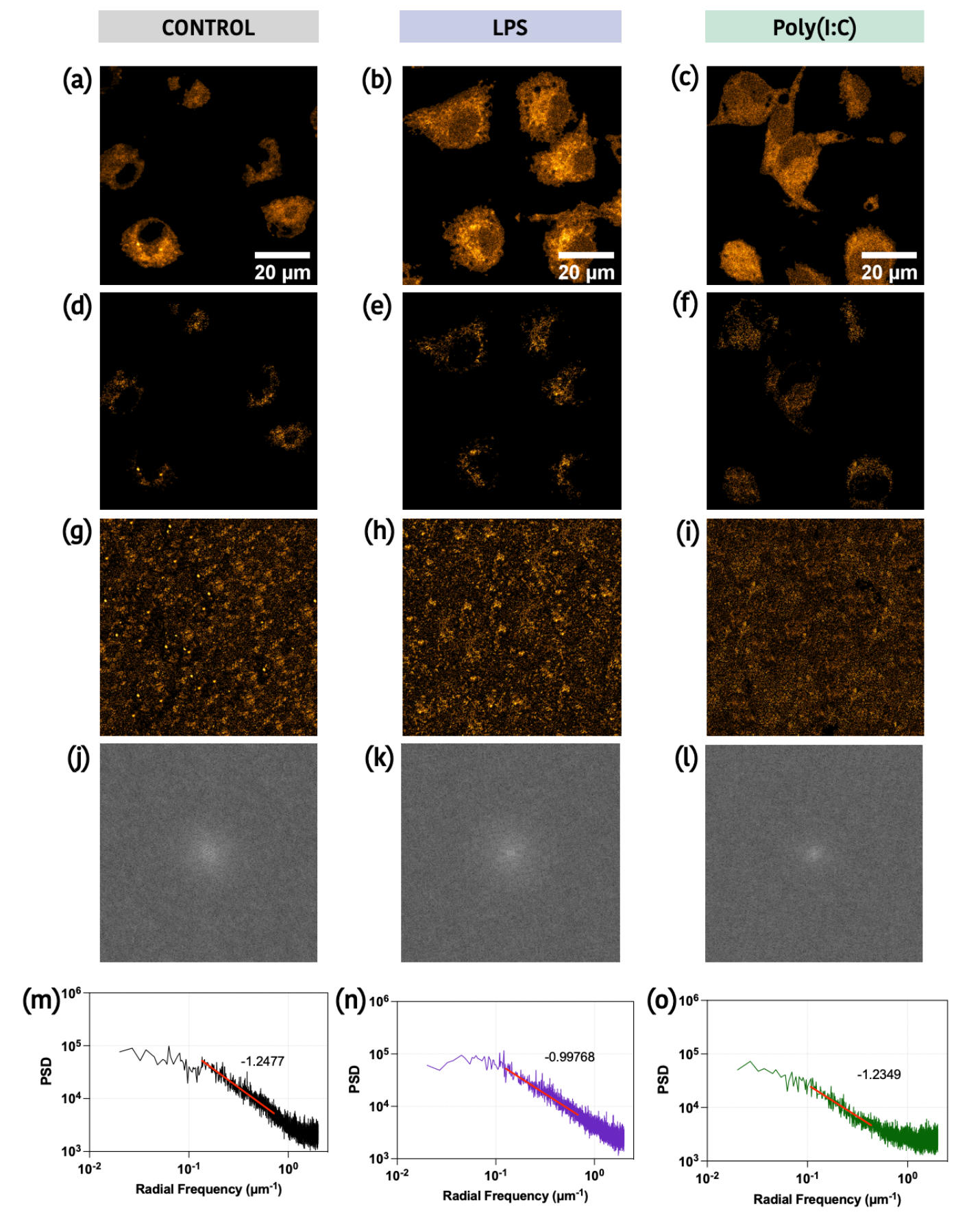


Figure S5: PSD analysis for BMM cells. Representative NAD(P)H-TPEF images (a-c) after thresholding is applied (d-f), after clone-stamping (g-i), and corresponding 2D Fourier transformed images (j-l). PSD spectra as a function spatial frequencies (m-o) for Control (a,d,g,j,m), LPS treated (b,e,h,k,n) and Poly(I:C) treated (c,f,i,l,o) cells.

***Summary of main results***

A summary of the changes found between a given treatment group (LPS or Poly(I:C)) and the control group is given in Table S6 below. This includes changes in the mean ORR, mean FAD-TPEF and NAD(P)H-TPEF intensities, spatial colocalization of FAD and NAD(P)H-TPEF intensities, and the FAD structural distance measurements. The direction (increase or decrease) of change and significance (represented by asterisks) are also provided.

Table S6. Changes in optical readouts in RAW264.7 cells and BMM under different metabolic stimuli compared to the control cells (* p ≤ 0.05, ** p ≤ 0.01, *** p ≤ 0.001, **** p ≤ 0.0001). The symbols ‘↑’, ‘↓’ and ‘-’ stand for ‘increase’, ‘decrease’ and ‘no change’ respectively.

|  |  | **Mean ORR** | **Mean FAD-TPEF intensity per cell** | **Mean NAD(P)H-TPEF intensity per cell** | **Spatial  Colocalization of FAD and NAD(P)H-TPEF intensities per cell** | **CM-FAD distance** | **FAD-FAD distance** |
| --- | --- | --- | --- | --- | --- | --- | --- |
| **RAW264.7** | **LPS** | ↓ **** | ↓ ** | - | ↓ **** | ↑* | ↑ ** |
|  | **Poly(I:C)** | - | - | - | ↑ ** | ↑** | - |
| **BMM** | **LPS** | - | ↓* | - | ↓ *** | ↑* | ↑ **** |
|  | **Poly(I:C)** | - | - | ↑** | - | ↑**** | ↑ ** |

**References**

1. N. Otsu. A threshold selection method from gray-level histograms. IEEE transactions on systems, man, and cybernetics, 9(1), 62-66. (1979). [↑](#endnote-ref-2)
2. Bolte, S., & Cordelières, F. P. A guided tour into subcellular colocalization analysis in light microscopy. *Journal of microscopy* **224**, 213-232 (2006). [↑](#endnote-ref-3)
3. Manders, E. M. M., Verbeek, F. J., & Aten, J. A. Measurement of co‐localization of objects in dual‐colour confocal images. *Journal of microscopy* **169**, 375-382 (1993). [↑](#endnote-ref-4)
4. Levitt, J. M. *et al*. Diagnostic cellular organization features extracted from autofluorescence images. *Optics Letters* **32**, 3305-3307, doi:10.1364/OL.32.003305 (2007). [↑](#endnote-ref-5)
5. Xylas, J., Quinn, K. P., Hunter, M. & Georgakoudi, I. Improved Fourier-based characterization of intracellular fractal features. *Optics express* **20**, 23442-23455 (2012). [↑](#endnote-ref-6)
6. Xylas, J. *et al.* Noninvasive assessment of mitochondrial organization in three-dimensional tissues reveals changes associated with cancer development. *International Journal of Cancer* **136**, 322-332, doi:<https://doi.org/10.1002/ijc.28992> (2015). [↑](#endnote-ref-7)
7. Lee, D. E. *et al*. A radiosensitizing inhibitor of HIF-1 alters the optical redox state of human lung cancer cells in vitro. *Scientific reports* **8**, 1-10 (2018). [↑](#endnote-ref-8)
